# Impact of methimazole‐induced hypothyroidism on postnatal swine

**DOI:** 10.14814/phy2.16007

**Published:** 2024-04-24

**Authors:** James C. Fazioli, Margaret K. Mulligan, Erin K. Ison, J. Alex Pasternak

**Affiliations:** ^1^ Department of Animal Science Purdue University West Lafayette Indiana USA

**Keywords:** goiter, hypothyroidism, methimazole, swine, thyroid Axis regulation

## Abstract

Thyroid hormones regulate metabolic rate, nutrient utilization, growth, and development. Swine are susceptible to thyroid suppression in response to disease or environmental conditions, but the physiological impact of such disruption has not been established. The objective of this study was to evaluate the impact of hypothyroidism induced with the antithyroid medication methimazole (MMI). 10 mg/kg MMI significantly decreased circulating triiodothyronine (T3) for the duration of treatment but had only a transient effect on circulating thyroxine (T4). Thyroid tissue weight was significantly increased by more than 3.5‐fold in response to MMI treatment. Histologically, the eosinophilic colloid was largely absent from the thyroid follicle which displayed a disorganized columnar epithelium consistent with goiter. MMI induced hypothyroidism has no effect on growth rate over 28 days. Hepatic expression of genes associated with thyroid metabolism (DIO1, DIO2, and DIO3), lipid utilization (CD36, FASN, and ACACA), apoptosis (TP53, PERP, SIVA1, and SFN) and proliferation (CDK1, CDK2, CDK4, and CDKN1A) were unaffected by treatment. Collectively these results demonstrate that MMI induces mild systemic hypothyroidism and pronounced goiter, indicating a strong homeostatic central regulation within the hypothalamic pituitary thyroid axis. This combined with limited peripheral effects, indicates resilience to hypothyroidism in modern swine.

## 1 INTRODUCTION

Collectively, thyroid diseases impact approximately 5% of the global population, encompassing over 200 million individuals worldwide (Zhang et al., 2023). Thyroid hormones play a crucial role in physiological function across nearly all organs and systems, including a primary role in metabolic regulation, and secondary roles in growth, differentiation and development (McAninch & Bianco, 2014). Despite well established central and peripheral mechanisms to maintain thyroid homeostasis, circulating levels of these hormones can be disrupted by a wide range of pathological processes and environmental factors (Shahid et al., 2024; Babić Leko et al., 2021). Thyroid disruption is a phenomenon observed across all species, but the physiological outcomes have been most extensively studied in rodents (Niedowicz et al., 2021) and sheep (Todini, 2007). Swine are increasingly recognized as an alternative model systems, which more accurately replicate human anatomy and physiology (Lunney et al., 2021; Wassen et al., 2004). Swine are a particularly relevant model for assessing hepatic function, a common implication of thyroid disease (Eberlova et al., 2020). Experiments in swine could elucidate the mechanisms behind thyroid disease and, in turn, enhance our comprehension of thyroid endocrinopathies in humans.

Thyroid hormones are characterized by a common two‐ring structure and are distinguished by the arrangement of 2–4 iodine molecules. Thyroxine (T4), the most abundant circulating hormone, is produced within the thyroid gland and secreted to peripheral tissues. Once in peripheral tissues, T4 can be converted into the bioactive Triiodothyronine (T3) through enzymatic deiodination of its outer ring. Alternatively, T4 can be converted to the largely inactive reverse T3 (rT3). Both rT3 and T3 can be further metabolized to one of multiple diiodothyronines (T2). In peripheral tissues, thyroid hormone is essential for biological function including nervous system development, metabolism, and growth (Shahid et al., n.d.; Cabello & Wrutniak, 1989; Mullur et al., 2014). Due to the importance of thyroid hormones, their homeostasis is meticulously controlled by the central regulatory mechanisms of the hypothalamic–pituitary‐thyroid (HPT) axis. Within this regulatory axis, thyrotropin‐releasing hormone (TRH) from the hypothalamus, signals the pituitary gland which in turn secretes thyroid‐stimulating hormone (TSH) which subsequently stimulates the thyroid to synthesize and secrete thyroid hormone. The resulting thyroid hormones then exhibit negative feedback on the production of additional TRH and TSH to maintain homeostasis. Outside of central thyroid hormone regulation, hormone metabolism mediated by a family of three iodothyronine deiodinase enzymes acts as a regulating mechanism in peripheral tissues through the progressive removal of iodine moieties (Sutcliffe & Harvey, 2015). Thus, the thyroid homeostasis is maintained through the collective interplay of the central HPT axis and peripheral deiodinase enzyme activity.

Clinical definitions of hypothyroidism involve a deficiency in the concentration of circulating thyroid hormone and often include goitrous pathology in response to the resulting increased pituitary TSH. An overabundance of TSH results in several distinctive histological features, including the transformation of thyroid follicle structure from a cuboidal to a columnar shape, a collapse of follicle structure, and a decreased presence of eosinophilic colloid in which the hormone is produced. Previous research has verified these histological changes in hypothyroid rodents and humans (Mizukami et al., 1993; Tsujio et al., 2007). Additionally, hypothyroidism has been demonstrated to impact metabolism, nutrient storage, lipid utilization, and decrease growth rate (Mullur et al., 2014; Schöne et al., 1997). Hypothyroidism poses additional risks during pregnancy, commonly leading to severe adverse impacts on both maternal and fetal health (Sahay & Vs, 2012). The profound consequences of hypothyroidism underscore the necessity for the use of precise disease models to gain a deeper understanding of its physiological effects and underlying mechanisms.

Anti‐thyroid compounds, including methimazole (MMI) and propythiouracil (PTU) are among the most commonly used medications in both human and veterinary medicine. Both compounds inhibit thyroid peroxidase (TPO) and thereby limit endogenous thyroid hormone production, but PTU exhibits additional effects on the peripheral deiodinase metabolism of thyroid hormone (Hassan et al., 2020). When given to a euthyroid individual, these compounds can be used to induce a controlled hypothyroid state, an approach that has been widely deployed in rodent models of hypothyroidism (Nambiar et al., 2014; Niedowicz et al., 2021). In addition to the hepatic effects of hypothyroidism, such studies have also associated the use of MMI with drug‐induced idiosyncratic liver injury (Nambiar et al., 2014), which is consistent with clinical observations in human (Gallelli et al., 2009; Gomez‐Peralta et al., 2018; Wang et al., 2014). To better understand this dual impact, we investigated the hepatic effects of MMI‐induced hypothyroidism using a postnatal porcine model.

## 2 MATERIALS AND METHODS

### 2.1 Animal model

All animal work was carried out in strict compliance with Institutional Animal Care and Use Committee regulations and approved by the Purdue University's Institutional Animal Care and Use Committee (IACUC #2103002122). A total of 12 healthy terminal cross (Landrace x Yorkshire dam, Duroc sire) castrated males (barrows), with an average weight of 9.0 kg were selected at 35 days of age. Animals were housed in individual pens within a semi controlled environment (18–22°C with regular cycles of artificial light in supplement of natural light) with ad libitum feed and water for the duration of the trial. Pigs were fed a standard nursery pig diet based on corn, soybean meal and dried distillers' grains and formulated with additional ingredients to meet established NRC requirements. Following a seven‐day acclimation period, *N* = 6 animals were randomly assigned to the hypothyroid treatment (TRT) with the remaining *N* = 6 serving as controls (CON). Hypothyroidism in the TRT group was induced with 10 mg/kg/day MMI (Sigma, Cat# M8506) administered orally as a 5 mg/mL solution in 50% corn syrup (Karo) with orange extract (Watkins) to enhance palatability. The daily dosage was manually administered by trained personnel via oral gavage to ensure complete ingestion of the treatment and with success indicated by the inclusion of red food coloring (McCormick). CON animals received an equivalent daily dose of corn syrup diluent with orange extract and green food coloring. Twice weekly both skin and rectal temperatures were taken, and body weights measured to adjust treatment dose. On days −3, 0, 4, 7, 11, 25, and 28 relative to the start of treatment, serum samples were collected and frozen at −20 ֯C for subsequent analysis of circulating thyroid hormone. All blood sampling was conducted between 9 and 11 am to reduce the influence of circadian rhythm on endocrine measurements and to minimize the effects of ambient conditions on temperature readings. All weaned barrows were humanely euthanized via captive bolt and exsanguination at 28 days following the initiation of treatment. Thyroid, thymus, heart, lungs, liver (LVR), spleen, kidneys, and longissimus dorsi (loin) muscle were extracted and weighed. Thyroid and LVR tissues were selected as the primary focus for analysis in the experiments. Digital images of the LVR including color and size standards were collected for subsequent morphometric analysis. Thyroid glands and a portion of the LVR were fixed in a 10% neutral buffered formalin and section of Liver and Ileum snap‐frozen in liquid nitrogen and stored at −80°C.

### 2.2 Thyroid hormone assay

The serum levels of total T4 and T3 were measured utilizing a commercially available Total T4 ELISA (Cat# 07M275A) and Total T3 Chemiluminescence Immunoassay (Cat# 07M175A) (MP Biomedicals, Solon, OH, USA). The assay procedures were followed as instructed from the manufacturer and followed as previously demonstrated in swine (Ison et al., 2023). In summary, 25 μL and 50 μL of serum were analyzed in duplicate for T4 and T3 respectively. Serum concentrations of T3 and T4 were determined for six time points (days −3, 0, 4, 7, 25, 28). Average inter‐ and intra‐ assay coefficients of variation were determined as 7.26% and 5.37% for T4 and 5.16% and 7.01% for T3 respectively. The limit of detection was previously established at 6.44 nmol/L for T4 and 0.19 nmol/L for T3.

### 2.3 RNA extraction and real time qPCR

Frozen LVR tissue samples were ground under liquid nitrogen into a fine powder using frozen mortar and pestles. RNA was extracted with TRIzol (Thermofisher Scientific, Waltham, USA) (Cat# 15596026) followed a double‐precipitation method as previously described (Mulligan et al., 2022). Contaminating DNA was removed with the TURBO DNase kit (Thermofisher Scientific, Cat# AM2238) following manufacturers instructions but with the addition of recombinant RNAse inhibitor (Thermofisher Scientific, Cat# N8080119). Concentration and purity of the isolated RNA was determined using a Nanodrop ND‐1000 (Thermo Fisher Scientific) and integrity verified through denaturing agarose gel electrophoresis (Kent‐Dennis et al., 2019). The High Capacity cDNA Reverse Transcription kit (Thermofisher Scientific, Cat# 4368814) was used to synthesize cDNA from an aliquot of 2 μg of purified total RNA. Gene‐specific primers for novel targets were designed against up‐to‐date mRNA sequences identified on RefSeq and targeting all predicted transcript variants. Where possible primers were designed to span exon‐exon junctions as identified by The BLAST‐like alignment tool (BLAT), relative to the *Sus Scrofa* genome assembly (SS11.1). Expression of a total of 14 genes was assessed, including five housekeeping genes and nine genes of interest which relate to cell cycle progression, apoptotic pathways, NAFLD disease, and deiodinase activity (Table 1). Primers were commercially synthesized and purified via standard desalting by IDT. The efficiency of each primer was determined to fall within 95%–105% of the expected range for each target and the melting curve was analyze to verify the lack of multiple amplicon products. qPCR was carried out in duplicate on 20 ng cDNA on the samples using Sso advanced universal sybr green supermix (BioRad, Hercules, USA) (Cat# 1725270) and CFX qPCR system (BioRad, Cat# 1855201). The stability of each housekeeping gene was assessed, and the two most stable genes for each tissue used to calculate a geometric mean with which to normalize the expression of the genes of interest. Fold changes relative to the mean expression of CON group samples were determined using the 2^−ΔΔCT^ method.

**TABLE 1:** Porcine specific primer sequences for qPCR.

	Gene symbol	Gene ID	Forward primer	Reverse primer	Amplicon length	Target RefSeq or source
Thyroid metabolism	DIO1	414,380	5’‐GACTTCATGCAAGGCAACAG‐3`	5’‐GGTCCTGGAGATTCTGGTGA‐3`	214	(Ison et al., 2022)
	DIO2	414,379	5’‐CTCGGTCATTCTCCTCAAGC‐3`	5’‐TCACCTGTTTGTAGGCATCG‐3`	140	(Pasternak et al., 2021)
	DIO3	414,378	5’‐CCTATCTGCGTGTCTGACGA‐3`	5’‐GCCTGCTTGAAGAAATCCAG‐3`	92	(Pasternak et al., 2021)
Apoptosis	TP53	397,276	5’‐ACAGTGACACGCTCTCCTGA‐3`	5’‐AGCTCAGAGGACAGCAGGTT‐3`	162	(Mulligan et al., 2022)
	PERP	100,513,507	5’‐TGGTGGAAGTGTTCTCAGGA‐3`	5’‐ACTCGCAGGAAGACAAGCAT‐3`	188	(Mulligan et al., 2022)
	SIVA1	110,258,343	5’‐CGCTACAGCTCAAGGTTCG‐3`	5’‐GGGTGGTCTTCTCGAAAATCT‐3`	93	(Mulligan et al., 2022)
	SFN	733,625	5’‐AGGAAGGCTCGGAAGAGAAG‐3`	5’‐ATGAGATGGGTGTTCAGCAAG‐3`	112	NM_001044564.1
Cell cycle Regulation	CDKN1A	100,152,215	5’‐CATGTGGACCTGTTGCTGTC‐3`	5’‐TTAGGGCTTCCTCTTGGAGA‐3`	168	(Pasternak et al., 2021)
	CDK1	100,155,762	5’‐CAGCTCGCTACTCAACTCCA‐3`	5’‐GAGTGCCCAAAGCTCTGAAA‐3`	135	(Pasternak et al., 2021)
	CDK2	100,154,715	5’‐CGGAGCTTGTTATCGCAAAT‐3`	5’‐AGGGGTAGGGTTCACAAAGG‐3`	143	(Pasternak et al., 2021)
	CDK4	100,144,492	5’‐TGGTTACAAGTGGTGGGACA‐3`	5’‐CCACAGAAGAGAGGCTTTCG‐3`	208	(Pasternak et al., 2021)
Lipid Utilization	CD36	733,702	5’‐ATGGTACAGATGCAGCCTCA‐3`	5’‐TTTCAGCTCCAAACACAGCA‐3`	108	NM_001044622.1
	FASN	397,561	5’‐GTTCATCTGCTCAGGGATGG‐3`	5’‐TGAGGCTCACGAAGGAAGAG‐3`	191	NM_001099930.1
	ACACA	397,324	5’‐CTCCAGGACAGCACAGATCA‐3`	5’‐CAGCATGTCAGAAGGCAGAG‐3`	247	NM_001114269.1
Reference genes	ACTB	414,396	5’‐CCAGCACGATGAAGATCAAG‐3`	5’‐AGTCCGCCTAGAAGCATTTG‐3`	171	(Pasternak et al., 2020)
	GAPDH	396,823	5’‐CCTGGAGAAACCTGCAAAAT‐3`	5’‐TTGACGAAGTGGTCGTTGAG‐3`	183	NM_001206359.1
	SDHA	780,433	5’‐CTACAAGGGGCAGGTTCTGA‐3`	5’‐AAGACAACGAGGTCCAGGAG‐3`	141	(Pasternak et al., 2016)
	STX5	100,628,048	5’‐TGCAGAGTCGTCAGAATGGA‐3`	5’‐CCAGGATTGTCAGCTTCTCC‐3`	144	(Pasternak et al., 2020)
	TBP	110,259,740	5’‐CTGAATGCTGAGGCGATTTC‐3`	5’‐GCTGTGGAGTCAGTCCTGTG‐3`	186	(Pasternak et al., 2020)

### 2.4 Histology

During tissue collection, isolated thyroids and samples of liver were fixed in a 10% neutral buffered formalin for a minimum of 48 h before processing and embedding in paraffin by the Purdue Histology Research Laboratory. The resulting histology blocks were sectioned on a HM 325 Rotary Microtome (Epredia, Kalamazoo, USA) (Cat# 902100) at 5 μm thickness and transferred to Superfrost Plus slides (Thermo Fisher Scientific, Waltham, MA, USA) (Cat# 12–550‐15). Slides were air‐dried overnight at room temperature before baking at 60°C for 20 min. Prior to staining slides were deparaffinized in xylene before rehydrating through decreasing concentration of ethanol. Duplicate sections of each thyroid were stained with hematoxylin and eosin for evaluation and imaging at multiple locations on each slide for a wholistic view of the tissue histopathology, using a Axio Imager.A2 microscope (ZEISS, Cat# 430005–9901‐000). In contrast three to five unique sections of liver were stained with 4′,6‐diamidino‐2‐phenylindole (DAPI) to visualize cell nuclei and Fluorescein conjugated wheat germ agglutinin (Vector Laboratoreis, Newak CA) (Cat# FL‐1021‐5) to visualize cell membranes. A minimum of nine fluorescent images of unique sections of tissue were captured for each sample, and processed through a custom semi‐automated macro for imageJ (Version 1.53 t) for quantitative analysis nuclei density and size. In short, WGA staining was used to manually define the largest possible artifact‐free area in each image, and this target region was then binarized for particle analysis. Values for threshold, particle size, and nuclei circularity were established in preliminary experiments and universally applied across all images. Results generated by the software were used to calculate nuclei density and average nuclei size as indirect measures of cellular hypertrophy and proliferation.

### 2.5 Liver colorimetric analysis

The anterior surface of individual livers was imaged along with a multicolor reference chip for calibration and scale. Colorimetric image analysis were carried out using imageJ (Version 1.53 t) with normalization and analysis conducted in R using a previously defined methodology (Inui et al., 1990; Kanamori et al., 2021) to determine the average values of the RGB color space.

### 2.6 Statistical analyses

All data analysis and statistics were carried out in R version 4.2.3 (R Core Team, 2019), with the addition of nlme (Pinheiro et al., 2019) and emmeans (Lenth, 2019) packages. Data for serum thyroid hormone, weight gain and body temperature were evaluated using a linear mixed effect model including treatment by day as fixed effects with repeat measures and Dunn–Šidák correction for multiple testing. Terminal organ weights were assessed using a standard linear model. Gene expression data was analyzed using the nonparametric Dunn's test, and is presented in the form of fold changes relative to the mean expression within the CON group using the 2‐ΔΔCT method. All data was reported as emmean values along with their corresponding 95% confidence intervals unless otherwise specified.

## 3 RESULTS

### 3.1 Circulating thyroid hormones

At trial day −3 before MMI treatment, there was no significant difference in the levels of circulating T3 and T4 thyroid hormone. Mean total T3 levels were 2.15 (1.58–2.73) nmol/L for CON and 1.96 (1.37–2.54) nmol/L (*p* = 0.609) for TRT while mean T4 levels were 107.2 (87.7–126.7) nmol/L for CON and 117.5 (97.8–137.2) nmol/L (*p* = 0.420) for TRT (Table 2). The impact of treatment on circulating thyroid hormone level was not uniform over time, with significant treatment by day interaction for T4 (*p* = 0.004) and a trending interaction for T3 (*p* = 0.055). Following MMI treatment, T3 levels in the TRT group decreased between day 0 and days 7–28, while T4 levels decreased between pre‐treatment and days 4–28 in. MMI treatment elicited a notable reduction in circulating T3 levels on days 4, 7, 25, and 28, reflecting a consistent temporal trend. Furthermore, a significant decrease in T4 levels was observed on days 7 and 25 in response to the treatment. Notably, there was no significant effect on days 4 or 28 following treatment for T4 levels, which may be attributed to an unexplained decline in T4 levels observed in the CON group on day 28.

**TABLE 2:** Estimated marginal means (95% confidence interval) for serum total T3 and T4 in nmol/L for control (CON) and MMI treated (TRT) swine on day −3,0,4,7,11,25 and 28 relative to the start of treatment.

	T3			T4		
Day	CON	TRT	Treatment *p* value^a^	CON	TRT	Treatment *p* value^a^
‐3	2.15 (1.58–2.73)	1.96 (1.37–2.54)^ab^	0.609	107.2 (87.7–126.7)^ab^	117.5 (97.8–137.2)^a^	0.430
0	2.42 (1.85–1.95)	2.53 (1.94–3.11)^a^	0.785	118.1 (98.6–137.5)^a^	104.4 (104.4–143.8)^a^	0.639
4	2.83 (2.26–3.41)	1.97 (1.39–2.55)^ab^	0. 041	96.5 (77.0–116.0)^ab^	77.1 (57.4–96.9)^b^	0.154
7	2.11 (1.54–2.68)	1.07 (0.48–1.65)^b^	0.018	74.4 (54.9–93.9)^b^	45.2 (25.4–64.9)^bc^	0.042
25	2.28 (1.71–2.85)	1.32 (0.73–1.90)^b^	0.026	87.4 (67.9–106.8)^ab^	41.0 (21.3–60.7)^c^	0.004
28	2.53 (1.96–3.11)	1.55 (0.97–2.13)^b^	0.023	78.0 (58.5–97.5)^b^	57.4 (37.6–77.1)^bc^	0.131

*Note*: Statistical differences over time for each analyte within treatment group are indicated with unique super scripts.

^a^ P values contrasts between TRT and CON within time point.

### 3.2 Thyroid histology

**FIGURE 1:**
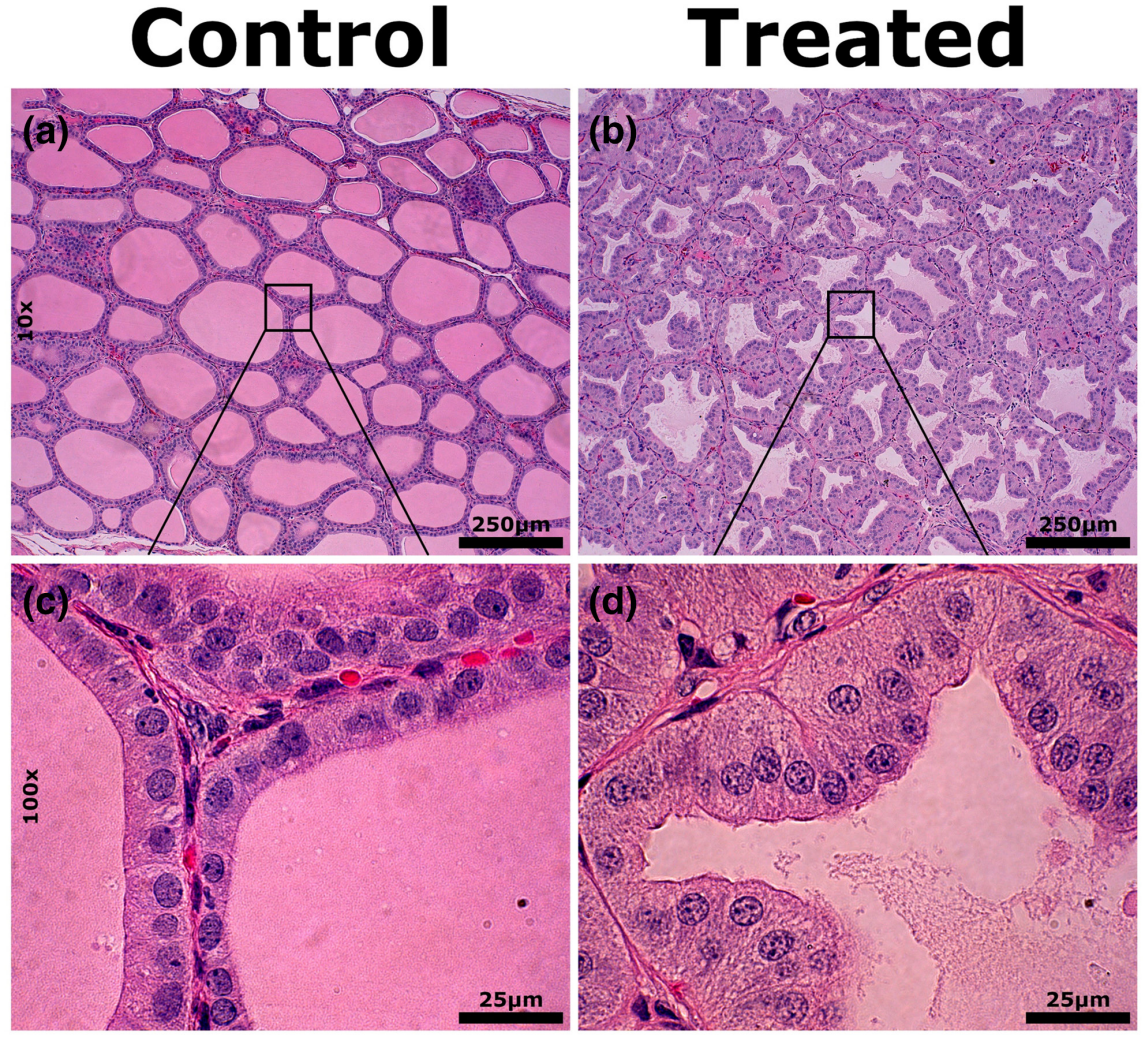
Representative 10x (A&B) and 100x (C&D) images of hematoxylin and eosin stained histological sections of thyroid tissue from control (A&C) and MMI treated (B&D) swine.

To verify the response to treatment, we next conducted histological analysis of thyroid sections at multiple magnifications (Figure 1). All TRT animals demonstrated histological changes to the thyroid consistent with hypothyroidism throughout the majority of the tissue. While dense and uniform in CON thyroid, the typically eosinophilic colloid in TRT thyroid follicles exhibited a clumped and granular appearance with minimal staining, indicative of impaired function. Thyroid follicle shape was perturbed, altered from well‐defined ovals in CON to shrunken, irregular shapes with a collapsed appearance in TRT. At higher magnification, we observed pronounced hyperplasia of the thyroid follicular epithelial cells of the TRT group, associated with a transition from a cuboidal to a columnar arrangement. The differences in thyroid histology between treatment groups was clear, supporting serum circulating hormone tests indicating goitrous pathology.

### 3.3 Effects of MMI on growth and thermoregulation

**FIGURE 2:**
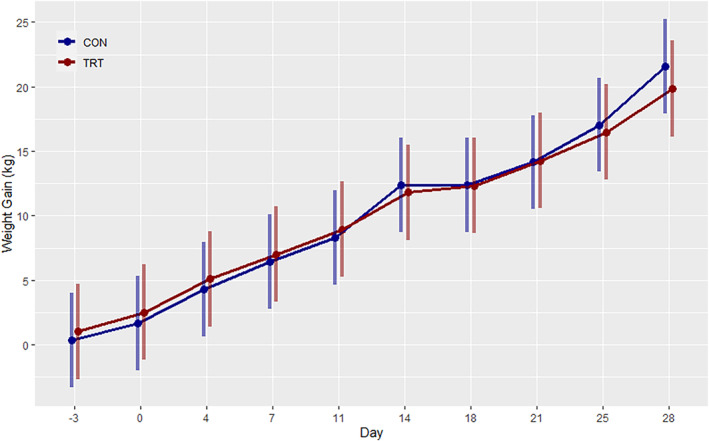
Estimated marginal means (95% confidence interval) for weight gain over the 28 day treatment period for Control.

Body weight increased significantly over time in all animals (*p* < 0.001), but MMI treatment had no significant impact (*p* = 0.967) on the weight gain over the course of the treatment period (Figure 2). Rectal and skin temperatures did not significantly differ between treatment groups at any time point measured in the study (data not shown). Upon completion of the 28‐day trial, thyroids from TRT animals exhibited a substantial 3.72‐fold increase in percentage of body weight (*p* < 0.001) relative to CON, consistent with the development of goiter. However, the remaining nine organs evaluated, showed no significant change in weight as a percentage of body weight relative to CON (Table 3).

**TABLE 3:** Estimated marginal means (95% confidence interval) for relative organ weights (g/kg body weight) after 28 days of either control (CON) and MMI treatment (TRT).

Tissue	CON	TRT	*p* value
Thyroid	0.123 (0.059–0.187)	0.458 (0.394–0.523)	<0.001
Adrenal	0.099 (0.07–0.128)	0.122 (0.096–0.149)	0.22
Spleen	2.331 (1.603–3.058)	1.791 (1.064–2.519)	0.28
Loin	12.319 (10.877–13.762)	12.741 (11.298–14.183)	0.66
Kidney	2.707 (2.068–3.347)	2.532 (1.892–3.171)	0.68
Brain	2.645 (1.782–3.508)	2.411 (1.548–3.275)	0.69
Heart	6.055 (4.053–8.057)	5.606 (3.604–7.608)	0.74
Liver	28.224 (20.462–35.986)	29.231 (21.469–36.994)	0.85
Lung	11.033 (8.087–13.98)	11.131 (8.184–14.077)	0.96
Thymus	2.493 (1.867–3.119)	2.49 (1.864–3.116)	0.99

### 3.4 Liver histology and color analysis

**FIGURE 3:**
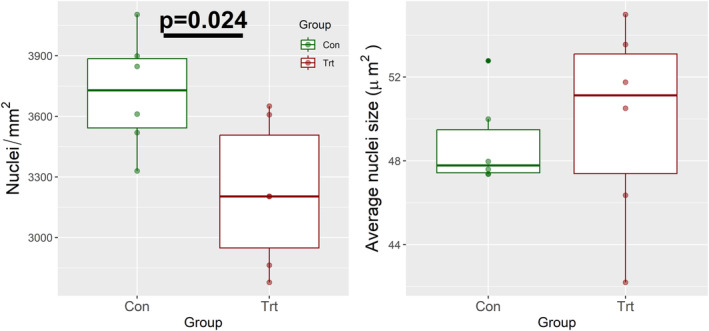
Estimated marginal means (95% confidence interval) for average nuclei density (nuclei/mm^2^) and average nuclei size (μm^2^) after 28 days of either control (CON) or MMI treatment (TRT).

To assess the potential impacts of treatment on fat accumulation in the liver, we employed colorometric surface analysis. A change in liver color could have indicated a difference in hepatic lipid utilization. After color normalization, no significant difference was found between the colors of CON and TRT livers (data not shown). ImageJ‐macro analysis of cell nuclei density (nuclei/mm^2^) and average nuclei area (μm^2^) for fluorescent stained slides to assess changes in cell and nuclei size. While there was no difference in average nuclei area (*p* = 0.637), there was a significant decrease in nuclei density (*p* = 0.024) from 3718 (3422–4014) (nuclei/mm2) in CON to 3218 (2922–3514) (nuclei/mm^2^) (Figure 3).

### 3.5 Effects of MMI on liver thyroid hormone metabolism gene expression

To understand the influence of MMI treatment on thyroid hormone metabolites, we aimed to examine the gene expression of deiodinases, which play a pivotal role in modulating thyroid hormones by facilitating their activation and deactivation. There was no difference in DIO1, DIO2, and DIO3 gene expression between evaluated liver tissue in the CON and TRT groups (Table 4). For DIO1, the gene expression remained consistent, with a fold change of 1.05 in CON and 0.85 in TRT, and no statistically significant difference (*p* = 0.436). Similarly, DIO2 exhibited comparable expression levels in both groups, with a fold change of 1.29 in CON and 1.11 in TRT, and no significant difference (*p* = 0.261). Likewise, the expression of DIO3 showed little variation, with a fold change of 1.05 in CON and 1.01 in TRT, and no statistically significant difference (*p* = 0.212).

**TABLE 4:** Median fold change for expression of genes associated with thyroid hormone metabolism, apoptosis, cell cycle regulation, and lipid utilization in hepatic tissue following 28 days of either control (CON) and MMI treatment (TRT).

		Fold change		
	Gene	CON	TRT	*p* Value
Thyroid hormone metabolism	DIO1	1.05	0.85	0.436
	DIO2	1.29	1.11	0.261
	DIO3	1.05	1.01	0.212
Apoptosis	TP53	1.08	1.12	0.374
	PERP	1.05	1.35	0.212
	SIVA1	1.04	1.35	0.055
	SFN	1.02	1.44	0.168
Cell Cycle regulation	CDKN1A	1.18	1.38	0.261
	CDK1	1.41	0.96	0.212
	CDK2	1.15	1.11	0.5
	CDK4	1.14	1.73	0.131
Lipid utilization	CD36	1.25	1.15	0.436
	FASN	1.09	1.29	0.436
	ACACA	1.18	0.96	0.374

### 3.6 Effects of MMI on liver apoptosis gene expression

To assess the potential of liver toxicity resulting from MMI treatment, we further examined hepatic expression of genes associated with the apoptosis including TP53, PERP, SIVA1, and SFN. Our investigation revealed no statistically significant differences in the expression of these apoptosis genes between the two groups (Table 4). For TP53, gene expression levels remained consistent, with a fold change of 1.08 in CON and 1.12 in TRT, indicating no significant difference (*p* = 0.374). Correspondingly, PERP displayed comparable expression levels in both groups, with a fold change of 1.05 in CON and 1.35 in TRT, and no statistically significant difference (*p* = 0.212). SIVA1 demonstrated a trending increase in expression within the TRT group with a fold change of 1.35 compared to 1.04 in CON, however the different did not reach statistical significance (*p* = 0.055). Expression of SFN demonstrated no statistically significant change in expression levels (*p* = 0.168) with a fold change of 1.02 in CON and 1.44 in TRT.

### 3.7 Effects of MMI on liver cell cycle regulation gene expression

To investigate the impact of MMI treatment on cell cycle regulation, we evaluated the expression of cell cycle suppressor gene CDKN1A and three cell cycle promoters (CDK1, CDK2, and CDK4) in the liver. Analysis of each cell cycle regulator yielded no significant differences in expression between treatment groups (Table 4). The expression of cell cycle suppressor CDKN1A established a fold chance of 1.18 in CON and 1.38 in TRT, causing no significant shift in expression with treatment (*p* = 0.261). Expression of CDK1, a cell cycle promoter responsible for the transition from S to G2 and G2 to M phases also saw no significant change (*p* = 0.212), with a fold change of 1.41 in CON and 0.96 in TRT. Evaluating CDK2, a cell cycle promoter required for the shift out of G1 and into the S phase of mitosis, demonstrated similar insignificant change in gene expression (*p* = 0.5) with a fold change of 1.15 in CON and 1.11 in TRT. Likewise, CDK4, the promoter composing the transition of cells from G1 to S phase in mitosis, exhibited no statistically significant alteration in expression, recording a fold change of 1.14 in CON and 1.73 in TRT (*p* = 0.131).

### 3.8 Effects of MMI on liver lipid utilization gene expression

To evaluate the likelihood of NAFLD, a disease characterized by the accumulation of excess fat in liver cells, we assessed the expression levels of the genes CD36, FASN, and ACACA which are associated with lipid utilization in the liver. None of the lipid utilization genes gauged demonstrated a significant difference in expression fold change in liver tissue (Table 4). The gene CD36 exhibited a fold change of 1.25 in CON and 1.15 in TRT, resulting in a statistically insignificant difference (*p* = 0.436) Expression of FASN was similar, with a 1.09‐fold change in CON and 1.29‐fold change in TRT yielding no significant result (*p* = 0.436). Finally, gene expression for ACACA remained similar with a fold change of 1.18 in CON and 0.96 in TRT, once again resulting in no significant change (*p* = 0.374).

## 4 DISCUSSION

Hypothyroidism is a common endocrine disruption characterized by a substantial decrease in circulating thyroid hormone, and when sustained, development of a goitrous thyroid. Experimentally, hypothyroidism has been induced in a number of species, including rats (Nambiar et al., 2014), chickens (Rosebrough et al., 2006) and swine (Schöne et al., 1997), using the antithyroid compound MMI. In the present study, MMI resulted in an overall reduction in serum T3 and T4 in juvenile swine over a 28‐day period. The combination of increased thyroid weight and goitrous pathology, including loss of eosinophilic colloid and pronounced perturbation in follicular epithelial cell shape and structure indicate the development of pronounced goiter. These histological changes in thyroid structure were consistent with pathological signs of goiter seen in rats exposed to MMI (Tsujio et al., 2007) as well as in hypothyroid human patients (Mizukami et al., 1993). Interestingly, the thyroid histology following MMI treatment differed from recent observation in fetal swine which showed a loss of eosinophilic colloid and collapse of thyroid follicle shape, but an undisturbed epithelial structure (Ison et al., 2023). We hypothesize this apparent difference in porcine thyroid response to MMI results from reduced pituitary capacity for TSH production during development of the HPT axis in the prenatal swine. Regardless, the combination of increased thyroid weight, reduced circulating thyroid hormone, and goitrous histology confirm the successful induction of mild systemic hypothyroidism.

While less substantial, a decrease in circulating T4 was also observed in untreated control swine, which is counter to the subtle increase previously observed in healthy post‐natal swine within a similar age range (Pasternak et al., 2021). However in the absence of goitrous pathology, we associate this apparent decrease with environmental factors such as temperature or humidity, which have been previously show to influence the thyroid system in swine (Macari et al., 1986). It is also worth noting that while MMI successfully induced hypothyroidism in the present study, circulating T3 and T4 levels (Ingram & Dauncey, 1986; Schöne et al., 1997) remained higher than previous studies from the mid 80's and 90's involving MMI treatment in swine. In one case this difference can be attributed to a dose of MMI 25 times greater than that used in the present study (Schöne et al., 1997). However, a study conducted in 1986 with swine of similar age and weight reports suppression of T4 hormone levels to undetectable levels, with a dose of just 2 mg/kg MMI per day (Ingram & Dauncey, 1986). Interestingly concentrations of thyroid hormone observed in control animals from the present study were also substantially higher than those reported over 25 years ago (Schöne et al., 1997; Spiegel & Blum, 1993), and more consistent with contemporary reports (Chapel et al., 2017). This apparent increase in resting thyroid hormone has been previously noted, and associated with intensive genetic selection of swine for lean growth rate in contemporary Landrace × Yorkshire cross barrows within a similar age range (Pasternak et al., 2021). Comparing the findings of our current study to historical models of MMI treatment suggests that genetic selection in swine has resulted in an increased resistance to drug‐induced thyroid hormone suppression over time. The results of the present study may imply that two more decades of selective breeding have not only elevated resting thyroid hormone levels in swine but have also conferred partial resistance to drug‐induced suppression of thyroid hormones. While no well validated assay for porcine TSH currently exists, development of such an assay would improve our understanding of the pituitary response to thyroid disruption in these highly selected animals.

Chronic overt hypothyroidism has long been associated with heightened sensitivity to cold temperatures and increased body weight in humans (Maushart et al., 2019; Sanyal & Raychaudhuri, 2016). However the impact of subclinical hypothyroidism on body weight remains controversial (Garin et al., 2014; Yan et al., 2022). In contrast, the mild systemic hypothyroidism induced in the present study we observed no significant change in body weight gain, or rectal/skin temp. These results must however be interpreted in context to the considerable growth rate observed in juvenile swine. The observed lean growth rate during the trial period would leave little energy for accumulation of body fat and increase in BMI observed in humans. In contrast previous studies in growing swine, associated hypothyroidism with reduced average daily gain (Schöne et al., 1997). Suppressed growth rate has also been reported in rats in response to severe hypothyroidism, but is linked to reduced daily feed intake (Alva‐Sánchez et al., 2012).

The biological activity of thyroid hormones depends on the number and location of their iodine moieties. Deiodinase enzymes mediate peripheral action by regulating the level of iodination on these hormones. Hypothyroidism has been noted to impact the expression of genes associated with deiodinases in rats, specifically through increased activation (DIO2) and decreased deactivation (DIO3) of thyroid hormone (Gereben et al., 2008; Silva & Matthews, 1984). Previous work in the fetal swine demonstrated altered hepatic deiodinase gene expression (DIO1, DIO2, DIO3) in response to severe MMI induced hypothyroidism (Ison et al., 2023). In contrast the present study found no significant change in hepatic deiodinase expression in response to mild systemic hypothyroidism. This result in combination with obvious signs of pronounced goiter, supports the theory that modern swine rely on strong central homeostatic mechanisms to resist HPT axis disruption rather than peripheral thyroid metabolism.

Thyroid hormones play an important role in the lipid metabolism of the liver. Previous research into the effects of hypothyroidism demonstrate lipid disorders, including elevated levels of serum cholesterol and the disruption in lipoproteins, both of which can be reversed through exogenous T4 hormone treatment (Duntas & Brenta, 2012; Sigal et al., 2011). Whether naturally or drug‐induced, hypothyroidism typically induces the accumulation of fat in the liver (Mavromati & Jornayvaz, 2021). We initially observed a significant decrease in nuclei density suggesting an increase in cellular volume consistent with lipid accumulation. However assessment of surface color, which is which is strongly correlated with liver steatosis in rodents and humans (Inui et al., 1990; Kanamori et al., 2021), suggested limited alteration in hepatic lipid content. In mice, mild hypothyroidism brought about by dietary iodine restriction, results in elevated hepatic expression of lipid utilization genes including ACACA, FASN, and CD36 (Ferrandino et al., 2017). In contrast, expression of these genes was not significantly affected by MMI treatment, suggesting MMI did not alter hepatic lipid utilization. We hypothesize that the steady and significant body weight gain of the post‐natal swine could lead to the allocation of any available nutrient to growth rather than storage, potentially preventing the development of a fatty liver through lipid accumulation.

Outside its experimental value, MMI is a widely used medication to counter hyperthyroidism in both humans and other animals. While an effective treatment, many of these studies have demonstrated drug‐induced cellular damage in a variety of tissues including the liver (Cano‐Europa et al., 2010; Cano‐Europa et al., 2011). Additionally, clinical research and case studies have indicated the development of idiosyncratic liver injury or other liver distress from MMI treatment (Gallelli et al., 2009). Controlled experiments in rats have demonstrated that the treatment of MMI can result in acute liver injury, consistent with drug‐related hepatotoxicity (Akai et al., 2016). Drug‐induced liver toxicity commonly results in inflammation and hepatocyte cell death, brought about by apoptosis or necrosis (Iorga et al., 2017). This effect may be common to anti‐thyroid medications, as experiments involving neo‐natal rats have identified increased expression of TP53, a transcription factor regulating apoptosis, upon treatment of PTU (Bunker et al., 2017). Thus we evaluated hepatic expression of TP53 along with downstream transcription targets that mediate apoptosis including PERP (Attardi et al., 2000), SFN (Wang et al., 2021) and SIVA1 (Singaravelu & Padanilam, 2011). The apoptotic pathway is inversely linked with cell proliferation, and PTU has also been shown to decrease expression of proliferating cell nuclear antigen (Bunker et al., 2017). We evaluated cyclic dependent kinase expression as well as CDKN1A, a potent cell cycle inhibitor known to be transcriptionally regulated by both TP53 and TGFB‐SMAD pathways, and again found no significant effect of MMI. Collectively, we find no evidence of hepatotoxicity in response this limited dose of MMI in swine. Conventional swine, such as the ones used in this study, are generally thought to reliably predict toxicity of various drugs and chemical in humans (Gad, 2000). Thus, additional studies using this porcine model are needed to evaluate MMI‐induced hepatotoxicity over a broader dose range.

## 5 CONCLUSIONS

The present study investigates the impacts of MMI treatment on thyroid function and related physiological responses in juvenile swine. Daily treatment of otherwise euthyroid swine with 10 mg/kg MMI induced mild systemic hypothyroidism, as evidenced by a significant decrease in circulating T3 and T4 levels. In spite of this mild response, substantial enlargement of the thyroid and histological signs of pronounced goiter, indicating a likelihood that the resistance to hypothyroidism was derived of central homeostatic mechanisms of the HPT axis. This mild hypothyroidism had minimal impact on hepatic thyroid metabolism, lipid accumulation, or drug induced toxicity. Additional studies on the effects of MMI in postnatal swine would be valuable to confirm heightened central resistance to hypothyroidism and to further assess potential hepatotoxic effects in this high value biomedical model.

## AUTHOR CONTRIBUTIONS

JAP conceived the study and obtained funding for the animal model which was developed and executed with the assistance of EKI and MKM. JCF conducted the molecular studies with assistance from MKM. JCF and JAP wrote the manuscript with input from MKM and EKI.

## CONFLICT OF INTEREST STATEMENT

The authors declare no conflict of interest.

## Data Availability

The data that support the findings of this study are available from the corresponding author upon reasonable request.
